# Supplementary material for: A measure of centrality in cyclic diffusion processes: Walk-betweenness
Source: PLoS One. 2021 Jan 28;16(1):e0245476. doi: 10.1371/journal.pone.0245476 (PMC7842912; doi:10.1371/journal.pone.0245476)
Supplement: Attachment — Submitted filename: Response to Reviewers.docx [file pone.0245476.s001.docx]

**Reviewer # 1**
*1) The authors tend to stick to the word 'diffusion' several qualifying adjectives, e.g. efficient, optimal, ... I think this may be slightly misleading. Rigorously, in physics, there is no such a thing as an efficient diffusion or an optimal one. Diffusion is diffusion, a very well defined dynamical process (here on networks), characterised by certain well defined features, e.g. the variance grows linearly with time. I think sometimes it would be better to replace the word diffusion with the nouns transport/movement/etc...which, by the way, are better qualified by the adjectives efficient and optimal.*

>>Thanks for your careful reading and suggestion. Sociologists used to use the term, ‘diffusion’, more broadly without exact definition across different contexts; for example, it includes social contagion, disease transmission and the diffusion of ideas, and innovation. That said, we acknowledge that the term could be misleading and as the reviewer suggested, we used the term ‘diffusion’ only when we refer to the general process and use other alternatives terms such as ‘spread’ or ‘transmission’ whenever necessary across the whole manuscript. The following paragraph shows the revision in the introduction section. You can confirm the corrections across the whole manuscript.

We propose a new bridging (brokerage) centrality measure for diverse types of networks that can accommodate an unlimited number of repeated interactions between nodes (actors). Most traditional measures are more appropriately suited for the transmission of valuable goods such as information and assume only optimal and efficient spreading between rational actors; however, our proposed measure is appropriate for disease transmission and web surfing where repeated back-and-forth interaction is the rule rather than the exception. In addition, this measure is well suited for the transmission of information of which the cost is negligible and of which the trustworthiness increases as a result of feedback provided during the back-and-forth transmission process (e.g., the spread of microfinance, fashion, and SNS usage).

*2) a Markov chain is a mathematical tool that can be used to model diffusion. Hence, the sentence 'Regular Markov Chain as a Diffusion Process' does not make much sense to me: a Markov chain is not a diffusion process.*

>>The point was fully accepted, and we removed the sub-title, “Regular Markov Chain as a Diffusion Process”. You can confirm this on page 12.

*3) what the author says about the model is fine in my opinion, though they could have slightly simplified the section if they had introduced the weaker concept of ergodic Markov chain (regular implies ergodic). Indeed, to have a stationary probability distribution, it is sufficient to have an ergodic chain.*

>>As far as we understand, regular chain is an ergodic chain that is not cyclic in mathematical terms. For example, if the transition matrix is $T=\left[\begin{matrix} 01 \\ 10 \end{matrix}\right]\text{, then }T^{2}=T^{4}=T^{6}=\left[\begin{matrix} 10 \\ 01 \end{matrix}\right]$ $\text{and, }T=T^{3}=T^{5}=\left[\begin{matrix} 01 \\ 10 \end{matrix}\right]$. In this case, *T* is ergodic, but not regular. Since we need a regular not ergodic chain to calculate the walk-betweeness, we would like to stick with the term, “regular” if it is fine with the reviewer. Anyhow, the term appears only twice in the manuscript and thus, if it is still the problem after this re-submission, we will change it without any problem.

*4) In 'Study 1. Diffusion of Syphilis in the Chicago Area': in the abstract and in the introduction the authors claim that the walk betweenness outperforms all the other centrality measures, though in this study no comparison has been made. I see here the necessity to compare the walk-betweenness, at least, with the measures mentioned in the introduction.*

>>Thanks for your feedback. Note that we have similar comments from Reviewer #2. As Reviewer #2 suggested, we added an analysis based on Newman betweenness score for the comparison: now, figure 4 contains an additional result from the Newman betweeness. As we highlighted in a new manuscript, our walk-betweenness measure reveals more hidden bridging communities than Newman betweenness measure. That said, we admit that it does not provide any conclusive evidence for which measure is better to identify the hidden bridging communities because bridging activity of a node cannot be observed directly. In order to judge which measure performs better empirically, we might need Syphilis incidents data at multiple time points so that we can compare the prediction from walk-betweeness with other alternatives, which is beyond our current study goal. Recall that we emphasized our walk-betweeness that allows multiple, repeated nodes or lines is also conceptually better than traditional centrality measures for the case of disease transmissions. We admit that our original manuscript did not provide enough discussion on this issue and we added more in the revised one. The newly added discussion is as follows.

Also, please note that while we conducted this job, we found a small encoding error in the matrix of communities for Syphilis transmission and corrected the error. As a result, now the figure 4 is a little bit different from the original and, also the cluster analysis produced seven clusters rather than fiver clusters. We double-checked the coding and we are confident that there is no coding error anymore.

That said, Panel B shows that Newman’s betweenness measure reveals fewer hidden bridging communities. This disparity comes from the difference in assumptions between two measures: Newman’s betweeness cancel out back-and-forth opposite transmission through a certain community to calculate net count while walk-betweeness count all the transmissions to obtain total count. We believe that this difference brings a bigger variance to walk-betweeness compared to Neman’s betweeness in general as shown in Figure 4, which could bring more hidden bridges.

Minor comments:

*5) - Pag. 6: in the sentence 'Paths rather than trails or walks are an appropriate diffusion process if the object of diffusion is a valuable resource and if the diffusion does not waste time or energy to repeat nodes or lines' there are two expressions which lead to some confusion, I believe.
Paths/Trails/Walks are not diffusion processes and diffusion is not an agent; I merely intend diffusion as a physical process, and as such it does not decide whether to 'waste' time and energy or not. One can base a centrality measure upon paths/walks/trails, and whether it is going to be a good measure it depends on the nature of the process they want to describe, for instance an optimised transport will agree with Freeman betweenness centrality and a diffusion with the walk-betweenness.*

>>Thanks for your comments. We simply agree with this point. Not-so-rare expressions in Sociology, which assumes agents, could be misleading to Physicists. We modified the sentence following Reviewer’s suggestion across the whole manuscript and the following is one example that clarify we assume human agents in the diffusion process.

If the object of diffusion is a valuable resource and if the agents of the diffusion do not want to waste time or energy to repeat nodes or lines, it would be natural to assume paths rather than trails or walks. Furthermore, shortest paths can be used to examine diffusion processes if the actors have adequate knowledge of the structure of the global network and are able to choose the shortest path. For example, when actors are seeking a piece of imminently necessary information, they prefer to traverse only the shortest paths; thus, people who are occupying bridging positions with high geodesic-betweenness scores obtain influential power.

*6) - pag. 11, first paragraph, end of second sentence: I guess it should be 'bridging potential' and not 'diffusion potential'.*

 >> Thanks. We modified the expression following your suggestion.

*7) - pag. 11, first paragraph, end of third sentence: typo 'meaninful' -> 'meaningful'.*

 >> We corrected the typo as you suggested.

*8) - pag. 13, line after '[Figure 2 about here]': 'each row of' should be replaced by 'each element of'.*

 >> We corrected the typo as you suggested.

*9) - pag. 14, first line: as it is not revelant for the discussion, I would not mention the word 'equilibrium'. Indeed, even though in mathematics the sense is slightly different, in physics, having a stationary distribution does not imply an equilibrium process, i.e.,* *stationary distribution may also represents out-of-equilibrium processes (think of a ring of states with links allowing only clockwise/anti-clockwise jumps).*

>> We agree with the point that having a stationary distribution does not imply an equilibrium process. As you suggested, we used the term ‘stationary distribution’ in place of the term ‘equilibrium distribution’.

*10) - Footnote 7: I believe this is an important part of the methods and should be included in the main text. Furthermore, there is a typo in the first line: 'the mean the first' -> 'the mean first'.*

>> As you suggested, the description in the Footnote 7 was moved to the main text. Also, we corrected the typo as you suggested.

*11) - Both in 'Study 1. Diffusion of Syphilis in the Chicago Area' and 'Study 2. Diffusion of Microfinance in Rural India': when the authors discuss how they handled the data they have, I believe they should be a bit more clear on how for instance they built the necessary matrices (introduced in ' MATERIALS AND METHODS') to perform the data analysis. For instance, it would be helpful to make references with the previous sections.*

>>We appreciate your suggestion. We added more detailed explanation about network data construction processes under newly added sub-heading, “Network data construction” now.

*Ideas and suggestions:*


*12) - Ranking nodes according to their bridging potential has been a productive research question even in the field of optimal percolation on networks (see for instance 'Nature 524, 65 (2015)' and 'Proc. Nat. Aca. Sci. 113, 12368 (2016)'). Theoretically speaking it would be nice to see a comparison (not necessarily here in this paper, but maybe in a future work) between walk-betweenness and other measures considered in optimal percolation.*

>>Thanks for your insightful suggestion. We agree that it will be important to compare our approach with the existing approaches in the field of optimal percolation on networks although as you said, it is beyond our current study. We, however, discussed this line of studies and pointed out some future directions as follows.

The identification of a set of nodes with high bridging potential, or say the “influential spreaders”, has been also widely studied in the field of optimal percolation on complex networks (Morone and Makse 2015; Braunstein et al 2016). For example, Morone and Makse (2015) propose a way to identify the minimal set of nodes to spread of information to the whole network by evaluating their role of maintaining the giant component after being removed based on the conception that the most influential nodes are the ones that enable the whole network to be connected. While our approach differs such measures in optimal percolation given that these measures are based on non-backtracking walks (i.e., a random walk that is not allowed to return back along the edge that it just traversed), future research is needed to compare our measure based on the back-and-forth transmission process against other measures considered in optimal percolation.

**Reviewer #2**

*1) The transition matrix T introduced at page 13 is the transition matrix associated to random walk and this should be mentioned. See Noh, Rieger, "Random walks on complex networks." Physical review letters 92.11 (2004): 118701.*

>> According to your kind suggestion, we mentioned the random walk and added the reference on page 13 in the revised manuscript.

*2) Page 16: the quantities z and d appearing in the equation are not defined. In general transmission correlation should be better explained, for instance explain the meaning of positive and negative correlation in terms of walk proximity.*

>> The definition was in footnote 7. We realized that, however, it could be confusing since the definition was hidden in a footnote. Thus, we mentioned this in the main text, now. Also, for a better explanation of the transmission correlation, we added the term, ‘walk proximity’ in two separate occurrences in the main text: page 16 and 20.

*3) It would be interesting to see the analogous of fig. 4 appear for other centralities, in particular Newman betweenness.*

# >>Thanks for your excellent suggestion, and we generated the same figure 4 for Newman betweenness measure. As we discussed briefly, our walk betweenness measure reveals more hidden bridging communities than Newman betweenness measure, which I believe highlights the contribution of our approach.

Also, please note that while we conducted this job, we found a small encoding error in the matrix of communities for Syphilis transmission and corrected the error. As a result, now the figure 4 is a little bit different from the original and, also the cluster analysis produced seven clusters rather than fiver clusters. We double-checked the coding and we are confident that there is no coding error anymore.

*4) Please provide more descriptive captions for both figures and tables, for instance mentioning which data set they refer to.*

>>Yes, we provided more descriptive captions for all figures and tables.

*5) Please provide a clear reference or repository for Syphilis data at the beginning of the relative section “Data”*

>> According to the suggestion, now we added more reference on page 24.

*6) I suggest an overall second read of the text in order to correct or rephrase some sentences, like for instance “Both data sets are secondary ones that had no personal identifiers from the beginning.”*

>> Yes, we agree that sentence reads awkward since it was written to show that we used the data without any confidentiality issue following Plos One’s guidelines right before the first submission. Now, we changed the problematic sentence, and improved other sentences as well across the whole manuscript.
