# Supplementary material for: A measure of centrality in cyclic diffusion processes: Walk-betweenness
Source: PLoS One. 2021 Jan 28;16(1):e0245476. doi: 10.1371/journal.pone.0245476 (PMC7842912; doi:10.1371/journal.pone.0245476)
Supplement: Attachment — Submitted filename: Response to Reviewers2.docx [file pone.0245476.s002.docx]

Both first-round reviewers were fully satisfied with our resubmitted manuscript and they agreed that the current manuscript is ready to be published.

However, Dr. Federico Botta, Academic Editor requested a section that explains the relationship of this work to one of previous manuscript and specify how this work further advances presentation of the walk betweenness measure. It was a quite valid concern and accordingly, we added a new paragraph to explain the difference between two papers and we are now confident that, it is obvious that our new manuscript advanced the discussion of walk betweeness in many ways.

**In your response to reviewers, please include a section that explains the relationship of this work to the previous manuscript, and specify how this work further advances presentation of the walk betweenness measure. Please clarify any relationship between the datasets used in the two works**.

Per your request, we added the following paragraph to the manuscript.

This measure was briefly introduced in a previous study (Youm 2010) without any elaborated mathematical explanation. The paper advances the discussion of walk-betweeness measure in three major ways. First, it provides the full mathematical equations behind the measure and lay out detailed calculations readers need to use to measure the walk-betweeness for themselves. Second, it introduces a new usage of walk-betweeness: a cluster analysis based on transmission coefficients. This cluster analysis can identify non-adjacent or distant neighborhoods who are close to each other in transmission paths. This could be essential to come up with a cooperative preventive strategy of local health-related organizations for transmittable diseases. Finally, it presents empirical tests of walk-betweeness measure by utilizing two new data sets: Syphilis transmission in Chicago area and microfinance diffusion in rural Indian villages.
